# ETV1 is a key regulator of enteroendocrine PYY production

**DOI:** 10.1242/dmm.052610

**Published:** 2025-12-12

**Authors:** Astrid M. Baattrup, Marianne Terndrup Pedersen, Stine L. Hansen, Martti Maimets, Fiona Gribble, Frank Reimann, Kim B. Jensen

**Affiliations:** ^1^ Novo Nordisk Foundation Center for Stem Cell Medicine (reNEW), Department of Biomedical Sciences, Faculty of Health and Medical Sciences, University of Copenhagen, Blegdamsvej 3B, 2200 Copenhagen, Denmark; ^2^ Biotech Research and Innovation Center (BRIC), University of Copenhagen, Ole Maaløes vej 5, 2200 Copenhagen, Denmark; ^3^ Institute of Metabolic Science, University of Cambridge, Hills Road, Cambridge CB2 0QQ, UK

**Keywords:** Enteroendocrine cells, GLP-1, PYY, ETV1, Intestinal organoids, L-cells

## Abstract

The intestine is a rich source of hormones that regulate metabolism. Among these are glucagon-like peptide-1 (GLP-1) and peptide YY (PYY), both expressed by L-cells. These hormones play important roles in promoting satiety; however, how they are regulated transcriptionally is not known. ETS variant transcription factor 1 (ETV1) is expressed by L-cells, but its function remains unknown. Here, we examined *Etv1* expression in single-cell RNA-sequencing (scRNA-seq) datasets from the mouse small intestine and from organoid cultures. To assess the functional role of ETV1, loss-of-function and overexpression experiments were performed in organoids. Gene expression was subsequently assessed with quantitative PCR and scRNA-seq. Our results confirmed *Etv1* enrichment in the L-cell lineage both *in vivo* and in organoids. Furthermore, mutations in ETV1 led to a decrease in *Pyy* expression levels with no effect on *Gcg* levels or on overall cell composition and organoid morphology. Moreover, overexpression of ETV1 led to a modest, but specific, increase in *Pyy* levels. We thus identified ETV1 as a regulator of *Pyy* expression, illustrating, for the first time, how specific hormones in the L-cell lineage are transcriptionally regulated.

## Teaser

**Summary:** The transcription factor ETV1 regulates the emergence of specific hormone-producing subtypes of enteroendocrine cells, in particular the PYY subset of the L-cell lineage.

## INTRODUCTION

Enteroendocrine cells (EECs) in the gastrointestinal tract constitute the largest endocrine organ of the human body, secreting more than 20 active hormones (Bany Bakar et al., 2023). Enteroendocrine (EE) hormones play vital roles in maintenance of metabolic control, and alterations in hormone expression and/or secretion are implicated in various diseases (Gribble and Reimann, 2019). In recent years, several studies have improved our understanding of EEC differentiation (Sanchez et al., 2022); however, many unknowns regarding the regulation of EEC differentiation and maturation remain.

EECs are positioned in the epithelium lining of the gastrointestinal tract and constitute ∼1% of the epithelial cells in the intestine. The luminal surface of the small intestine is folded into crypts and villi, and, like other mature cells in the intestinal epithelium, EECs differentiate from intestinal stem cells (ISCs) residing at the bottom of intestinal crypts (Barker et al., 2007; Cheng and Leblond, 1974). The progeny from ISCs is continuously pushed out of the stem cell niche. Once outside, cells differentiate either along the absorptive lineage into enterocytes or along the secretory lineage into Paneth cells, goblet cells, tuft cells or EECs (Snippert et al., 2010).

EECs have traditionally been divided into subtypes based on detected hormones. These subtypes include enterochromaffin cells expressing serotonin, D-cells [somatostatin (SST)], X-cells [ghrelin (GHRL)], K-cells [glucose-dependent insulinotropic polypeptide (GIP)], S-cells [secretin (SCT)], L-cells (GLP-1, PYY), I-cells [cholecystokinin (CCK)] and N-cells [neurotensin (NTS)] (Sjölund et al., 1983). The density of different EEC subtypes varies along the length of the intestinal tract. K-cells are abundant in the proximal small intestine, whereas L-cells exhibit an increasing prevalence from the proximal to the distal small intestine and colon (Parker et al., 2009; Roberts et al., 2019; Polak and Bloom, 1982). Moreover, hormone expression patterns differ along the crypt–villus axis, with GLP-1-expressing cells predominantly found in crypt domains, while others, such as NTS-expressing cells, are primarily observed in villus domains (Grunddal et al., 2016). The first classifications into EEC subtypes were based on the notion that each subtype expressed one hormone (Solcia et al., 1981). However, it has since been shown that there is a large degree of hormonal co-expression within EECs, and a level of plasticity between the originally defined subtypes as individual cells change hormone expression along the crypt–villus axis (Beumer et al., 2018; Egerod et al., 2012; Habib et al., 2012; Gehart et al., 2019). Recent work has suggested that, instead of the original eight EEC subtypes, only five different EE lineages exist, as L-, I- and N-cells are all part of the same lineage (Gehart et al., 2019).

L-cells were first characterised by expression of several small peptide hormones, all encoded by the *GCG* gene, of which GLP-1 has achieved most attention (Holst, 2007). Subsequent studies showed co-expression with the hormone PYY (Böttcher et al., 1984). Expression of PYY is highest in the distal part of the intestine and is detected later than GCG during L-cell maturation (Gehart et al., 2019; Adrian et al., 1985). PYY is an anorexigenic hormone and functions in the central nervous system, where it promotes long-term satiety (Gribble and Reimann, 2019). Together with GLP-1, PYY is also an important mediator of the ‘ileal brake’, a mechanism that slows down gastric emptying and intestinal motility, leading to a reduction in food intake. Moreover, PYY has been suggested to promote pancreatic β-cell survival, while GLP-1 is an incretin hormone, promoting insulin secretion from the pancreas (Andersen et al., 2018; Lafferty et al., 2018). These functions make PYY and GLP-1 attractive therapeutic molecules for conditions such as diabetes and obesity. Analogues of GLP-1 are used therapeutically for treatment of diabetes and obesity (Nauck et al., 2021), while treatment with PYY analogues as well as combination treatment with GLP-1 and PYY analogues have been evaluated in ongoing clinical trials (Müller et al., 2022). Despite the obvious therapeutic value of PYY and GLP-1, we have little knowledge about regulation of L-cell differentiation and hormone expression.

Our current knowledge related to control of EEC identity and hormone expression is mostly limited to a range of key specifiers of the EEC fate determination from intestinal stem cells. Neurogenin 3 (NEUROG3) expression is necessary for EEC differentiation, as *Neurog3* knockout mice lack all EECs (Jenny et al., 2002). Several other transcription factors have been implicated at different timepoints during EEC differentiation, some of which have been reported to be specific for one or more EEC subtypes (Gehart et al., 2019; Du et al., 2012; Li et al., 2011; Piccand et al., 2019; Desai et al., 2008; Larsson et al., 1998; Beucher et al., 2012; Li et al., 2019; Terry et al., 2014). However, although the function of some of these regulators has been described, others remain unexplored – in particular, their role in specifying hormone expression. ETV1 is part of the ETS family of transcription factors (Sharrocks, 2001). It has an ETS DNA-binding domain as well as two transactivating domains, and it has been implicated in neuronal differentiation and endocrine development in the pancreas (Kobberup et al., 2007; Benitez et al., 2014; Arber et al., 2000; Janknecht, 1996). Interestingly, several other key regulators of EECs are shared with neuronal cells (Hayashi et al., 2023). *Etv1* has been shown to be enriched in the L-cell lineage (Habib et al., 2012; Gehart et al., 2019), yet it remains unclear whether ETV1 plays a role in EECs and hormone expression.

The study of L-cells and other EECs is complicated by their scarcity *in vivo*. The development of organoid culture systems for primary epithelial cells has greatly improved our ability to study these rare cell types (Sato et al., 2009). Furthermore, organoids derived from the small intestine have been shown to retain their regional identity, which includes differences in the types of hormones expressed (Basak et al., 2017; Middendorp et al., 2014; Maimets et al., 2022).

In this study, we characterised the role of ETV1 within the intestinal epithelium with a particular focus on the EEC lineages. We analysed tissue samples and utilised organoid cultures for functional studies. Using loss-of-function mutations and overexpression (OE) in organoid models, we outlined the role of ETV1 in hormone expression. In agreement with others, we found that *Etv1* is enriched in L-cells. Our results further demonstrate that ETV1 specifically regulates *Pyy*, with little or no effect on other gut hormones. This is one of the first examples of a hormone-specific transcription factor, providing new insight into how hormone production is dynamically regulated within the intestine.

## RESULTS

**Fig. 1.**
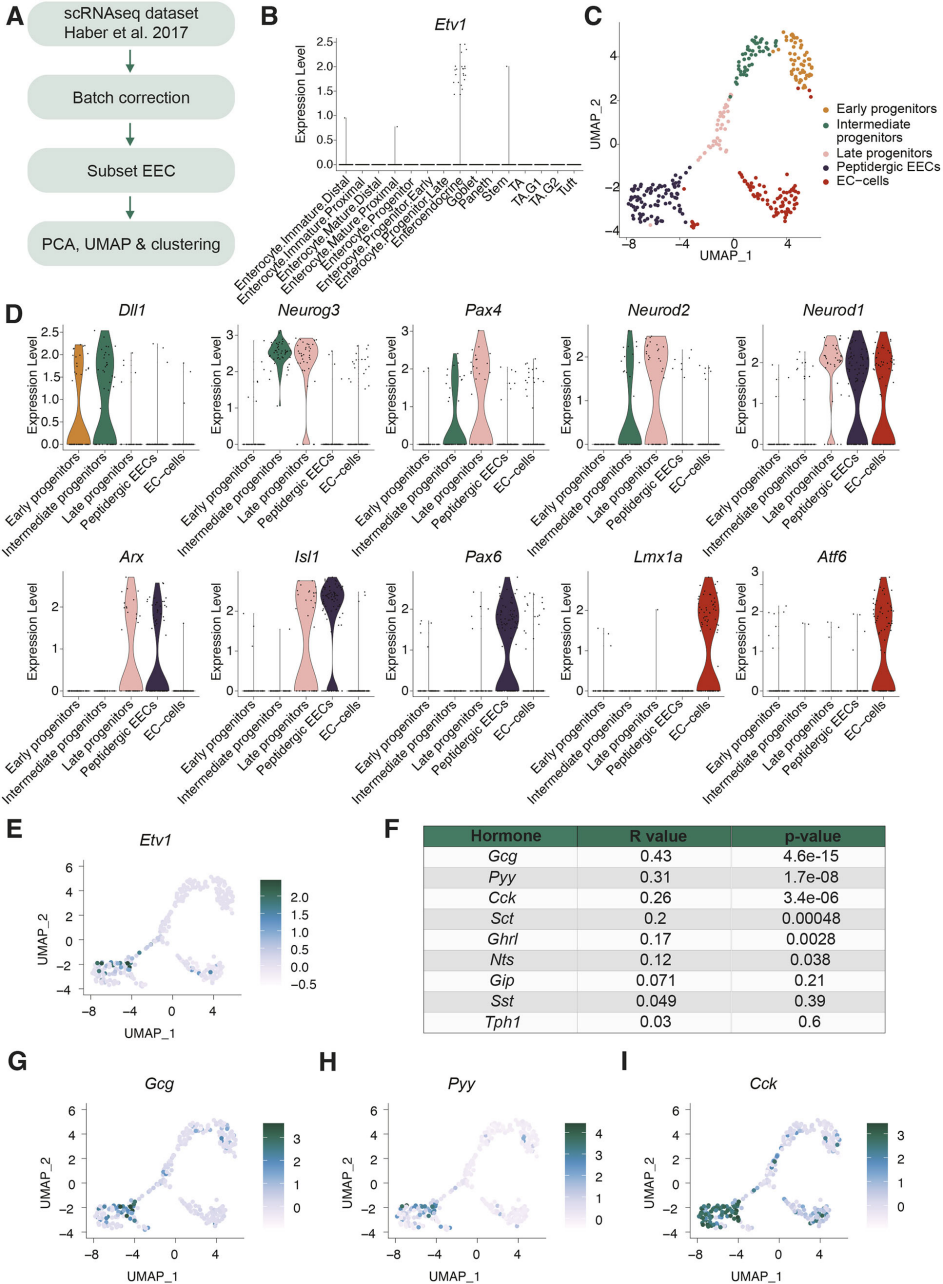
***Etv1* expression is most strongly correlated with expression of *Gcg*, *Pyy* and *Cck in vivo*.** (A) Workflow for analysis of enteroendocrine cells (EECs) from the single-cell RNA-sequencing (scRNA-seq) dataset published by Haber et al. (2017). PCA, principal component analysis; UMAP, Uniform Manifold Approximation and Projection. (B) Expression of *Etv1* across different cell type clusters (original cell type annotation). TA, transit amplifying. (C) UMAP plot following unsupervised clustering of EECs from mouse small intestine. Clusters are annotated based on expression of known marker genes with a temporal expression pattern during EEC differentiation. EC, enterochromaffin cell. (D) Violin plots showing expression of selected genes involved in EEC differentiation across cell type clusters of EECs from mouse small intestine. (E) UMAP plot showing expression levels of *Etv1* in EECs from mouse small intestine. (F) Correlation between *Etv1* expression and expression of different enteroendocrine (EE) hormones. R-value=Pearson correlation coefficient. (G-I) UMAP plots showing expression of *Gcg* (G), *Pyy* (H) and *Cck* (I) in EECs from mouse small intestine.

To characterise *Etv1* expression in the mouse small intestinal epithelium, we analysed a published single-cell RNA-sequencing (scRNA-seq) dataset (Fig. 1A; Fig. S1A,B) (Haber et al., 2017). Using the reported cell type annotation, we found that *Etv1* was almost exclusively expressed by EECs (Fig. 1B). We therefore focused our analysis on this cluster, and performed principal component analysis (PCA), Uniform Manifold Approximation and Projection (UMAP) and unsupervised clustering of EECs (Fig. 1A,C; Fig. S1C). Clusters were annotated based on marker expression reported to have a temporal expression pattern during EE differentiation (Fig. 1D) (Gehart et al., 2019). In accordance with previous findings, *Dll1* was defined as an early marker of secretory differentiation, followed, in sequential order, by expression of *Neurog3*, *Pax4*, *Neurod2* and *Neurod1*. Further, in line with other studies, we found that the peptidergic EECs expressed *Arx*, *Isl1* and *Pax6* and formed a cluster distinct from enterochromaffin cells that were positive for *Lmx1a* and *Atf6* (Gehart et al., 2019; Piccand et al., 2019; Gross et al., 2016)*. Etv1* was mainly detected in peptidergic EECs, but also in a small subset of enterochromaffin cells (Fig. 1E). In the peptidergic EECs, *Etv1* expression showed the strongest correlation with expression of *Gcg*, *Pyy* and *Cck* (Fig. 1F-I; Fig. S1D). We validated co-expression of *Etv1* and *Pyy* with fluorescent *in situ* hybridisation (ISH). As expected, *Pyy*-expressing cells were not present in the proximal small intestine but increased along the length of the intestine, with the highest numbers observed in the distal small intestine and large intestine. In all segments of the intestine in which *Pyy*-expressing cells were present, we observed cells that co-express *Etv1* (Fig. S2A,B).

**Fig. 2.**
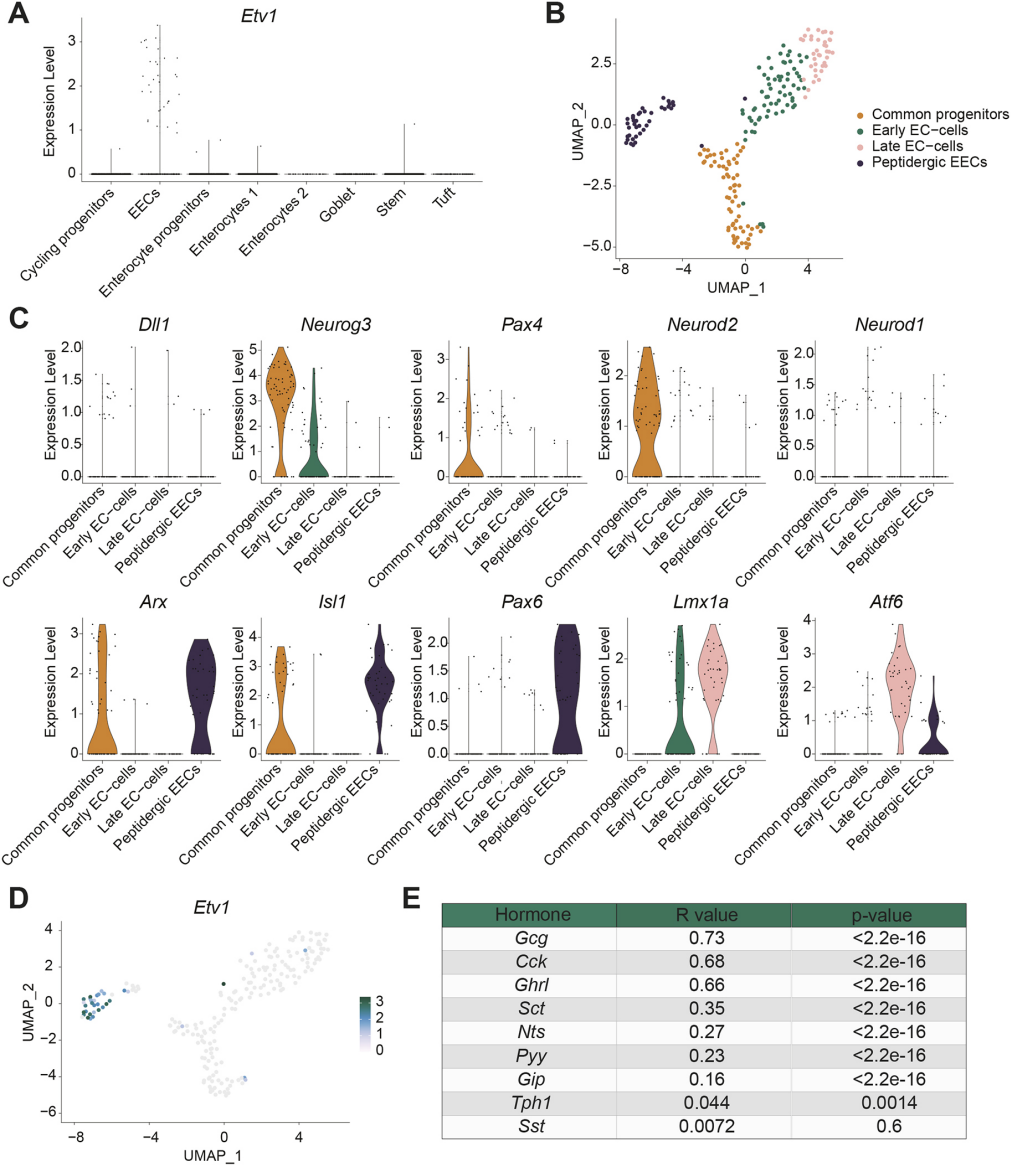
**EEC differentiation and *Etv1* expression in organoid cultures resemble *in vivo* observations.** (A) Expression of *Etv1* across different cell type clusters (cell type annotation from Hansen et al., 2023). (B) UMAP plot following unsupervised clustering of EECs from organoid cultures. Clusters are annotated based on expression of known marker genes with a temporal expression pattern during EEC differentiation. (C) Violin plots showing expression of selected transcription factors involved in EEC differentiation across cell type clusters of EECs in organoids. (D) UMAP plot showing expression levels of *Etv1* in EECs from organoids. (E) Correlation between *Etv1* expression and expression of different EE hormones in organoid cultures. R-value=Pearson correlation coefficient.

Based on the unique expression pattern of *Etv1*, we hypothesised that it was implicated in specification of the L-cell lineage. To address this question, we turned to the organoid model (Sato et al., 2009). To confirm that EEC differentiation and the *Etv1* expression pattern in organoids resemble the observations from *in vivo* studies, we analysed EECs and *Etv1* in a published scRNA-seq dataset from mouse intestinal organoids (Hansen et al., 2023). Aligned with observations *in vivo*, *Etv1* expression was only detected in EECs, which again could be separated into peptidergic EECs and enterochromaffin cells (Fig. 2A,B). Supporting the validity of the organoid model for studying EECs, *Neurog3*, *Neurod2* and *Pax4* were detected in progenitor cells, while expression of *Arx*, *Isl1* and *Pax6* was restricted to peptidergic EECs and *Lmx1a* and *Atf6* to enterochromaffin cells (Fig. 2C). Again, *Etv1* was detected almost exclusively in mature peptidergic EECs (Fig. 2D). Interestingly, we only detected low levels of *Pyy* and *Nts* (Fig. S3A). It is known that EECs expressing these hormones are normally detected on the upper-villus domain, and it is possible that organoid cultures have difficulties fully recapitulating later differentiation stages (Grunddal et al., 2016). Correlation studies illustrated again the strongest correlation between *Etv1* and *Gcg* as well as *Cck* expression (Fig. 2E; Fig. S3B). In conclusion, our analyses demonstrated that EE differentiation and *Etv1* expression in organoids resemble the *in vivo* observations and thereby validated the organoid system as a useful model for interrogating the role of ETV1 in EECs.

**Fig. 3.**
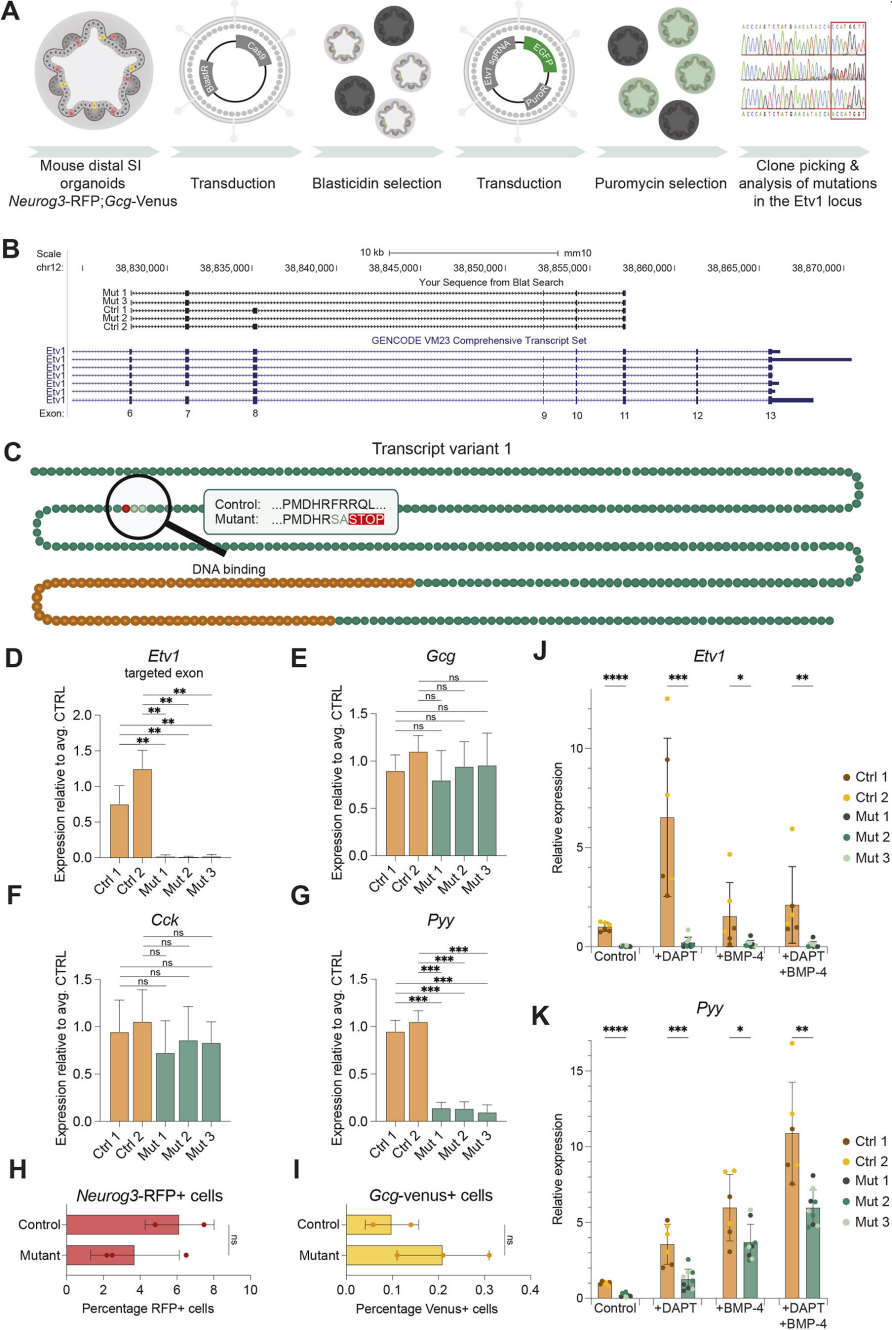
***Etv1* mutant cultures have reduced *Pyy* expression.** (A) Strategy for generation of *Etv1* mutant organoid lines. Ngn3, *Neurog3*. Created in BioRender by Jensen Team (2025). https://BioRender.com/eqtfeop. This figure was sublicensed under CC-BY 4.0 terms. (B) Amplified and sequenced *Etv1* cDNA aligned to the *Etv1* gene using the BLAT alignment tool. Screenshot downloaded from http://genome.ucsc.edu. (C) ETV1 protein (transcript variant 1). One dot corresponds to one amino acid (AA). Skipping of exon 8 changes AA 186-187 from phenylalanine (F) and arginine (R) to serine (S) and alanine (A) and introduces a premature stop codon after AA187, resulting in a protein that lacks the DNA binding domain (orange dots). (D-G) Expression of *Etv1* (D), *Gcg* (E), *Cck* (F) and *Pyy* (G) in control and *Etv1* mutant organoid cultures. The *Etv1* reverse primer is located within exon 8. Expression is normalised to expression of *Gapdh.* Error bars indicate s.d. (*n*=3). Significance was evaluated with an unpaired two-tailed *t*-test. CTRL, control. (H,I) Percentage of Neurog3-RFP^+^ (H) and Gcg-Venus^+^ (I) cells in control (two lines) and *Etv1* mutant (three lines) organoid cultures assessed by flow cytometry. Error bars indicate s.d. Significance was evaluated with an unpaired two-tailed *t*-test. (J,K) Expression of *Etv1* (J) and *Pyy* (K) in control and *Etv1* mutant organoid cultures treated for 3 days with or without 10 µg DAPT and/or 20 ng/ml BMP-4. Significance was evaluated with an unpaired two-tailed *t*-test. ns, not significant; *P<0.05, **P<0.01, ***P<0.001, ****P<0.0001.

To explore the role of ETV1 in EECs, we mutated the *Etv1* locus using CRISPR/Cas9 in organoids derived from the ileum of *Neurog3*-RFP;*Gcg*-Venus mice (Fig. 3A) (Kim et al., 2015; Reimann et al., 2008). We targeted exon 8, as it is part of all known *Etv1* splice variants, and alternative splicing skipping exon 8 results in a truncated protein. Following lentiviral transduction with sgRNA and *Cas9*, three mutant clonal organoid lines, carrying different genomic alterations in the *Etv1* locus, were chosen for further characterisation. Two additional lines with no alterations were used as controls in subsequent experiments. Sequencing of *Etv1* cDNA from the three mutant lines showed that they all skipped exon 8, introducing a stop codon after amino acid 187 (Fig. 3B; Fig. S4A,B). The resulting protein is missing the DNA-binding domain and is therefore lacking its repressive or activating function on direct targets (Fig. 3C; Fig. S4C). *Etv1* mutant organoids were morphologically indistinguishable from control organoids (Fig. S4D). Despite the introduction of the premature stop codon, *Etv1* mRNA was not degraded by nonsense-mediated decay, as RNA could be detected from exons downstream of exon 8, but not from exon 8 (Fig. 3D; Fig. S5A). In agreement with *Etv1* being expressed at later stages of EEC development, we did not observe a change in *Neurog3* expression in the *Etv1* mutant organoid lines (Fig. S5B).

To test the effect of the *Etv1* mutation on lineage specification and maturation, we went on to analyse gene expression of hormones in the mutant lines (Fig. 3E-G; Fig. S5C-F). Notably, we observed a very prominent reduction in the levels of *Pyy* (90%) in the mutant lines, whereas the levels of *Gcg* and *Cck* were unaffected (Fig. 3E-G). Interestingly, we also found a tendency towards reduction in expression of *Nts* and *Sct* – two hormones that are both known to be co-expressed with *Pyy* – in the mutant *Etv1* organoid lines (Fig. S5C,D) (Grunddal et al., 2016; Egerod et al., 2012; Habib et al., 2012; Gehart et al., 2019). In agreement with our quantitative PCR (qPCR) data, we found no difference in the fraction of *Neurog3*-RFP^+^ and *Gcg*-Venus^+^ cells in mutant and control organoids (Fig. 3H,I; Fig. S5G). Differentiation into the secretory lineage is increased upon inhibition of Notch signalling, while increased BMP signalling has been reported to increase expression of upper-villus hormones, such as PYY (Beumer et al., 2018; VanDussen and Samuelson, 2010). Thus, to increase the number of *Pyy*-expressing cells, we cultured control and mutant ETV1 organoids in the presence of DAPT and/or BMP-4 (Fig. S5H). After 3 days we assessed expression of *Etv1*, *Pyy*, *Gcg* and *Cck* (Fig. 3J,K; Fig. S5I,J). In all conditions, we observed reduced expression of *Pyy* in the mutant cultures compared to that in controls (Fig. 3K).

**Fig. 4.**
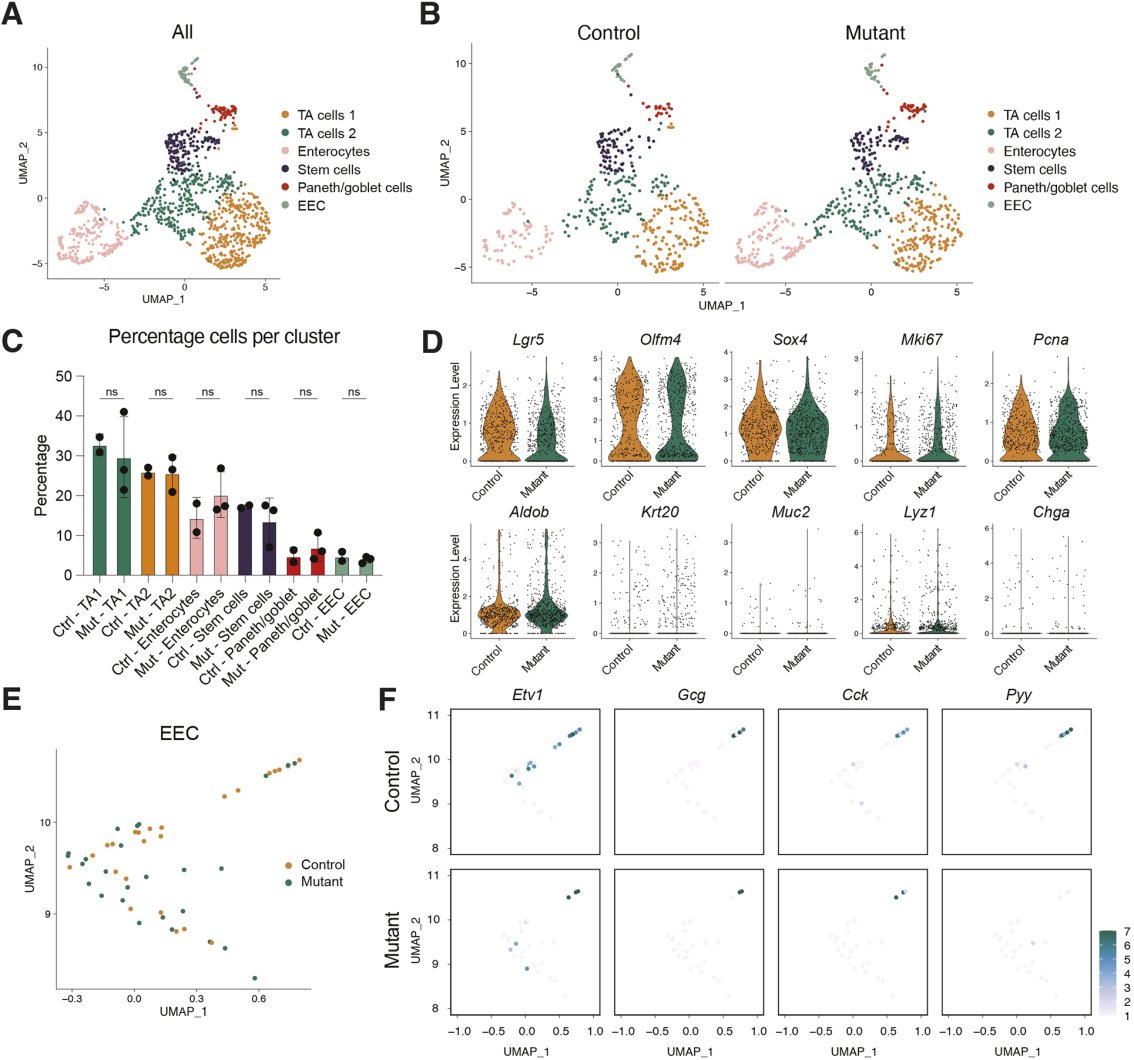
***Etv1* mutant organoids show no overall changes in cell type composition but lack EECs with high *Pyy* expression.** (A) UMAP plot of cells from both control (two lines) and *Etv1* mutant (three lines) organoids following scRNA-seq (1158 cells in total). Cell types are annotated based on expression of known marker genes (Fig. S4A). (B) UMAP plot of cells from control (left) and *Etv1* mutant (right) organoids (control, 494 cells; *Etv1* mutant, 664 cells). (C) Percentage of cells found in each of the identified cell clusters in control (two lines) and *Etv1* mutant (three lines) organoids. Error bars indicate s.d. Significance was evaluated with an unpaired two-tailed *t*-test. ns, not significant. (D) Violin plots showing expression levels of known cell type and proliferation marker genes in control (orange) and *Etv1* mutant (green) organoids. (E) UMAP plot showing EECs in control (orange) and *Etv1* mutant (green) organoids. (F) UMAP plot showing expression levels of *Etv1*, *Gcg*, *Cck* and *Pyy* in EECs from control (top row) and *Etv1* mutant (bottom row) organoids.

To further examine the effect of mutating *Etv1* on hormone expression, we went on to perform scRNA-seq on the mutant and control lines. Cells from the datasets were clustered and annotated based on canonical marker gene expression identifying the expected cell types (Fig. 4A; Fig. S6A,B). In agreement with *Etv1* only being expressed in the EECs, mutation of *Etv1* did not cause any overall changes in the proportion of the major cell types or in the expression of various cell and proliferation marker genes (Fig. 4B-D). Focusing the analysis on the EEC lineages recapitulated the qPCR data. We observed a pronounced absence of cells expressing *Pyy* in the mutant dataset, whereas *Gcg* and *Cck* were detected in both control and *Etv1* mutant organoids (Fig. 4E,F). Furthermore, in agreement with our qPCR data, cells expressing high *Nts* levels were absent in the dataset from mutant organoids, while *Sct* expression was reduced in mutant organoids (Fig. S6C). Collectively, these results demonstrated that, although there was a strong correlation between *Etv1* and *Gcg* expression *in vivo* and *in vitro*, ETV1 specifically regulates late-stage EEC L-cell maturation and *Pyy* expression. The fact that we saw no change in *Gcg* expression suggests that ETV1 is not involved in initial specification into the L-cell lineage.

**Fig. 5.**
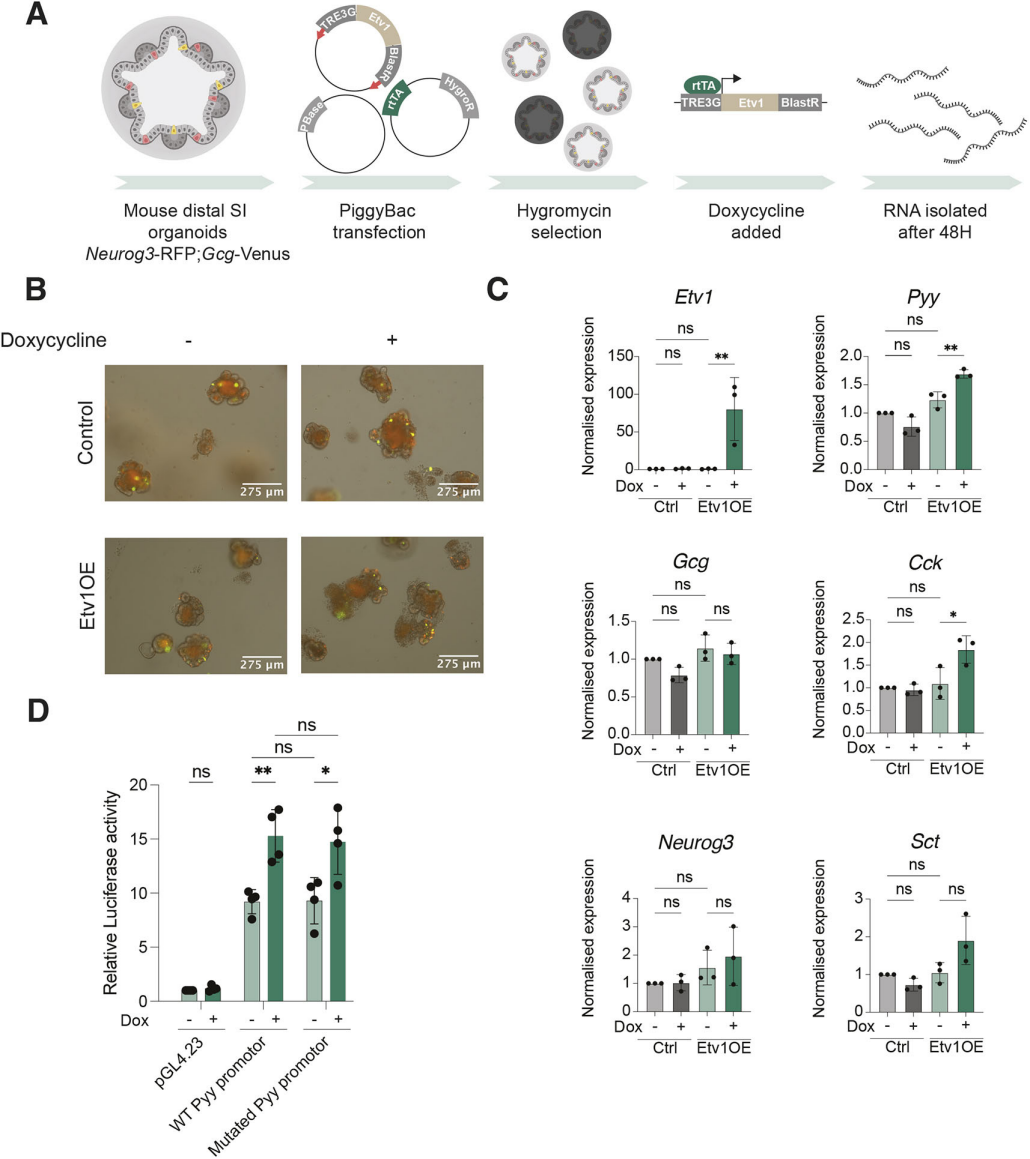
***Etv1* overexpression increases expression of *Pyy* and *Cck*, but not *Gcg*.** (A) Strategy for generation of *Etv1*-overexpressing (*Etv1*OE) organoids. SI, small intestine. Created in BioRender by Jensen Team (2025). https://BioRender.com/eqtfeop. This figure was sublicensed under CC-BY 4.0 terms. (B) Images of control and *Etv1*OE organoids with and without 48 h of doxycycline treatment. Scale bars: 275 µm. Organoids were derived from a *Neurog3*-RFP;*Gcg*-Venus mouse (Kim et al., 2015; Reimann et al., 2008). (C) Expression of *Etv1*, *Pyy*, *Gcg*, *Cck*, *Sct* and *Ngn3* (*Neurog3*) in control and *Etv1*OE organoid cultures with and without 48 h doxycycline treatment. Error bars indicate s.d. (*n*=3). Expression is normalised to expression of *36B4* (*Rplp0*). Significance was evaluated with a one-way ANOVA. (D) Luciferase activity in inducible *Etv1*OE HEK293 cells transfected with a pGL4.23 vector containing either a wild-type (PyyProm_WT) or mutated (PyyProm_MUT) version of a 517 bp region upstream of *Pyy* covering two putative ETV1 binding sites (Fig. S7A). Luciferase activity was normalised to the activity in HEK293 cells transfected with a pGL4.23 vector without any insert. Where indicated, cells were treated for 24 h with doxycycline (1 mg/ml). Error bars indicate s.d. (*n*=4). Significance was evaluated with an unpaired two-tailed *t*-test. ns, not significant; *P<0.05, **P<0.01.

To further investigate whether ETV1 could have a specific role in regulating *Pyy* expression, we turned to a system that enabled us to overexpress ETV1 in an inducible manner (*Etv1*OE) in the organoid cultures (Fig. 5A). Organoids transfected with PiggyBac transposase (PBase) and the reverse-tetracycline TransActivator (rtTA) were used as controls. *Etv1*OE and control organoids were treated with doxycycline for 48 h to induce *Etv1* expression, which did not lead to morphological changes (Fig. 5B). In line with the reduced expression of *Pyy* in the mutant organoids, we observed a modest, but reproducible, increase in the levels of *Pyy* in the *Etv1*OE cultures, again with no change in *Gcg* expression (Fig. 5C). Interestingly, we also observed an increase in *Cck* expression in *Etv1*OE cultures, whereas we did not observe any change in expression in the *Etv1* mutants. Collectively, we found that ETV1 overexpression confirms a role for ETV1 in regulating *Pyy* expression.

To investigate whether ETV1 directly regulates elements within the *Pyy* promoter, we analysed the upstream sequences of PYY for predicted ETV transcription factor binding sites. Here, we identified two putative ETV1 binding sites (Fig. S7A), with predicted binding sites for several other ETV family members also found upstream of *Pyy*. We did, however, confirm that ETV1 is the only ETV specifically upregulated in the L-cell lineage (Fig. S7B,C). Next, we generated two reporter constructs containing the genomic region upstream of *Pyy* spanning the two putative ETV1 binding sites placed upstream of *luc2*. One construct contained the wild-type (WT) *Pyy* promotor sequence, while the putative ETV1 binding sites were mutated in the second construct (Fig. S7A). To assess the effect of ETV1 expression on Luc2 expression, HEK293 cells in which ETV1 overexpression could be induced by doxycycline were transfected with two different luciferase constructs. Results showed increased luciferase activity upon ETV1 overexpression, indicating that ETV1 binds to the genomic region upstream of *Pyy* and increases gene expression (Fig. 5D). We saw no difference between the WT and mutated sequence, suggesting that either the induced mutations were insufficient to prevent ETV1 binding or ETV1 binds in a different location. Collectively, these results suggest that ETV1 can regulate PYY expression, through direct binding to a sequence upstream of *Pyy*.

In conclusion, we show that ETV1 regulates the emergence of specific hormone-producing subtypes of EECs, in particular the PYY subset of the L-cell lineage, without any major effects on differentiation of intestinal stem cells into the EE lineage.

## DISCUSSION

Given the role of EEC hormones in maintenance of metabolic control, improving our knowledge of how their expression are regulated holds great therapeutic potential. In this study, we identify ETV1 as a regulator of EECs expressing PYY. Based on a careful characterisation of the intestinal epithelial cells focusing on the EEC lineages, we show that *Etv1* is expressed primarily in the L-cell lineage. Functionally, we show that ETV1 does not affect general intestinal stem cell differentiation even upon overexpression, but rather has a very specific role in regulating the onset of *Pyy* expression. Upon overexpression of *Etv1*, we only observe a slight increase in *Pyy* expression. This might, in part, be explained by the organoid lines not being independently generated, but could also indicate that *Etv1* expression is not able to drive differentiation into the L-cell lineage. Thus, the observed increase in *Pyy* expression might only originate from the very small subset of organoid cells, belonging to the L-cell lineage. The fact that we see no change in *Gcg* expression upon *Etv1* mutation or overexpression supports that allocation into the L-cell lineage is not dependent on *Etv1*. Interestingly, ETV1 expression in human small intestine and colon is, similarly to our findings in mice, confined to EECs and co-expressed with GCG, PYY and CCK, suggesting that ETV1 in human might play a role similar to that we describe for mouse (Burclaff et al., 2022).

Previous studies have also reported *Etv1* expression in EECs (Habib et al., 2012; Gehart et al., 2019). Time-resolved analysis of differentiation within the EE lineage following cells from the onset of *Neurog3* expression revealed *Etv1* as a late factor emerging ∼40-50 h after the onset of differentiation (Gehart et al., 2019). Notably, the increase in *Etv1* expression in this dataset preceded an increase in *Pyy* expression. These findings are well aligned with our functional data demonstrating that ETV1 plays a key role in regulating *Pyy* expression. Given that both mutation and overexpression of *Etv1* affect *Pyy* expression, it is plausible that ETV1 regulates *Pyy* expression directly, which is further supported by our luciferase data. To validate these findings, chromatin immunoprecipitation (ChIP) experiments would be needed, but this is complicated by technical limitations, as L-cells are too scarce for most ChIP experiments.

In addition to our observations relating to *Pyy*, we observed a trend towards decreased *Nts* and *Sct* expression in the *Etv1* mutants, as well as an increase in *Cck* expression and a trend towards increased *Sct* expression in the *Etv1*OE cultures. *Nts*, *Sct* and *Cck* were reported to be co-expressed with *Pyy* and *Gcg* in a common EEC lineage (Gehart et al., 2019). Of these hormones, expression of *Gcg* was shown to increase first during EEC maturation, whereas expression of the other hormones, similarly to *Pyy*, increased slightly later. Based on this, it could be speculated that, in addition to a specific role in regulating *Pyy* expression, ETV1 might also play a broader role in the overall maturation state of L-cells. Moreover, a reduction in *Nts* expression in *Etv1* mutants would provide experimental support for L- and N-cells belonging to the same EEC lineage. A role for *Etv1* in regulating *Nts* and *Sct* expression, might also explain the rare presence of *Etv1*-expressing cells in the proximal small intestine, where *Pyy* is not expressed (Fig. S2A). Alternatively, it is entirely possible that *Etv1* plays other roles in the intestinal epithelium in addition to its role in regulating *Pyy* expression.

Transcriptional profiling identified *Etv1* as one of the genes being strongly reduced in L-cells from mice fed a high-fat diet for 16 weeks (Richards et al., 2016). Notably, other downregulated genes included *Pyy*, *Nts*, *Sct* and *Cck*. It is tempting to speculate that downregulation of ETV1 could be at least partially responsible for the deregulated hormone expression, but further studies are required to delineate how ETV1 contributes to the complex regulation of EEC functions upon dietary changes.

Diabetes and obesity are growing health problems, which when left untreated can lead to a range of comorbidities. At present therapies rely on the use of EE hormone analogues, mostly focusing on GLP-1 analogues (Nauck et al., 2021). Given the role of GLP-1 and other hormones as regulators of our metabolism, understanding how individual hormones are regulated from a molecular perspective is of vital importance. Similarly to GLP-1, PYY regulates appetite by binding to receptors in the brain, providing a long-lasting sense of satiety, which highlights its therapeutic potential. However, many aspects of PYY signalling remain unknown. For instance, it was recently shown that, in addition to its role as a hormone, PYY might also function as an antimicrobial peptide (Pierre et al., 2023). Therefore, increased knowledge of the different PYY functions have the potential to expand its therapeutic scope beyond treatment of diabetes and obesity. In this context, our identification of ETV1 as a regulator of *Pyy* transcription provides an important insight into how endogenous PYY expression potentially can be regulated therapeutically. Here, it is worth noting that ETV1 is subjected to multiple post-translational modifications, which affect its half-life and DNA-binding affinity and thus potentially could be targeted (Janknecht, 2003; Wu and Janknecht, 2002; Vitari et al., 2011; Goel and Janknecht, 2003; Bosc et al., 2001).

A comprehensive understanding of how specification of the EEC subpopulations is controlled, as well as how expression of individual hormones is regulated, would provide us with a vast number of possibilities to control and modulate our metabolism. This would not only have implications for the treatment of diabetes and obesity but could potentially also be used to treat reduced appetite, for instance in patients receiving chemotherapy or older adults. As such, the findings presented in this study constitute a step towards achieving this goal.

## MATERIALS AND METHODS

### Mice

*Neurog3*-RFP;*Gcg*-Venus mice were used for organoid derivation (Kim et al., 2015; Reimann et al., 2008). All animal procedures were approved by The Danish Animal Inspectorate.

### Organoid culture establishment and maintenance

Intestinal crypts were isolated from the distal 6 cm of the small intestine of *Neurog3*-RFP;*Gcg*-Venus mice. The intestine was flushed with PBS and cut open, and villi were removed by softly scraping with a glass slide. The intestine was cut into smaller pieces and incubated in PBS with 2 mM EDTA for 1 h. After vigorously shaking, crypts were filtered using a 70 µm cell strainer and seeded in 30 µl drops [2/3 Matrigel (MG; Corning) and 1/3 medium] in a 48-well plate. Advanced DMEM/F12 (Gibco) containing penicillin/streptomycin (50 μg/ml/50 μg/ml; Gibco), Glutamax (1×; Gibco), Hepes (10 mM; Gibco), B27 supplement w/o vitamin A (1×; Gibco), recombinant human EGF (50 ng/ml; Peprotech), recombinant murine noggin (100 ng/ml; Peprotech), R-spondin conditioned medium (100 µl/ml) and N-acetyl-L-cysteine (1 mM; Sigma-Aldrich) (ENR medium) supplemented with ROCK inhibitor (Y-27632; ROCKi) (10 µM; Sigma-Aldrich) was added after the MG solidified (20-30 min), and organoids were incubated at 37°C and 5% CO_2_. ROCKi was removed from the medium after 2 days. Organoid cultures were passaged by mechanical dissociation using a p1000 and p200 pipette into fragments. Organoids were passaged every 4-7 days and maintained in ENR medium. For generation of clonal organoid lines, organoids were passaged as single cells. 2 days prior to passage, CHIR99021 (3 µM; Calbiochem) and nicotinamide (10 mM; Sigma-Aldrich) were added to the organoid medium (ENR-CN). Organoids were collected and dissociated into single cells by incubation in TrypLE Express (Gibco) for 5-10 min at 37C. Single cells were seeded in 2/3 MG and 1/3 ENR medium. ENR-CN medium supplemented with ROCKi was added after 20 min. 2 days after passage, medium was changed to ENR medium. When indicated, DAPT (10 µM; Sigma-Aldrich) and/or recombinant human BMP-4 (20 ng/ml; R&D Systems) was added to organoid cultures on day 3 after passage. For conditions treated with BMP-4, noggin was omitted from the medium. Organoids were collected on day 6 (3 days of treatment), and gene expression was assessed using qPCR. All organoid lines were regularly tested for contamination.

### Reagent concentration

#### Generation of *Etv1* mutant cultures

*Etv1* gRNA (*AGTCTATGAACATACCACCA*) and a scramble gRNA were cloned into CRISPR.SFFV.EGFP (Addgene plasmid #57827), in which *Cas9* had been replaced by *Pac*. For lentiviral production, one 10-cm dish with 80% confluent HEK293 cells was transfected with 10 µg transfer vector (pEtv1-gRNA-Pac, pScramble-gRNA-Pac or lentiCas9-Blast (Addgene plasmid #52962), 7.5 μg packaging vector and 3 μg envelope vector in 0.25 M Cacl_2_ mixed with one volume 2× HEPES buffered saline. Medium was changed after 8 h, and medium containing viral particles was collected 2 and 3 days after transfection. Collected medium was ultracentrifuged and resuspended in 100 µl ENR medium. 4 days prior to transduction, ENR-CN medium was added to the organoid medium. Organoids from three wells were collected per transduction and mechanically dissociated into small fragments. Fragments were resuspended in 100 µl ENR-CN medium supplemented with polybrene (1:1000) and ROCKi, and 12.5 µl virus was added. Cells with virus were spun for 1 h at 600 ***g*** at 32°C, followed by 6 h incubation at 37°C, after which they were washed and seeded in 2/3 MG and 1/3 ENR medium. ENR-CN medium+ROCKi was added after 20 min. Transduced cells were selected with blasticidin (5 μg/μl; Thermo Fisher Scientific) and/or puromycin (2 μg/μl; Sigma-Aldrich) 2 days after transduction. ENR-CN medium+ROCKi was replaced with ENR medium after 4 days. Following selection, organoids were passaged as single cells, and single organoids were picked to generate clonal lines. DNA was isolated from one well (48-well plate) using a DNeasy Blood & Tissue Kit (Qiagen) according to manufacturer's instructions. Primers were designed using NCBI Primer-BLAST and used to amplify the genomic region around the expected Cas9 cut site within the *Etv1* locus (98°C for 30 s; 29 cycles of 98°C for 10 s, 55°C for 30 s, 72°C for 45 s; 72°C for 10 min). Primer products were purified with a MinElute PCR Purification Kit (Qiagen) and sequenced with Sanger sequencing. Similarly, *Etv1* cDNA from the clonal lines was amplified (98°C for 3 min; 31 cycles of 98°C for 10 s, 55°C for 30 s, 72°C for 45 s; 72°C for 10 min), purified and sequenced. Sanger sequencing was done by GATC Services. Primer sequences for amplification of *Etv1* genomic DNA (gDNA) and cDNA were as follows: *Etv1* gDNA fwd, 5′-GACGTTTAAAGTGCACACTAGCA-3′; *Etv1* gDNA rev, 5′-ACCCATGCCCTCAACTGTAG-3′; *Etv1* cDNA fwd, 5′-GTTCAGAACTCGGGTCTGCT-3′; *Etv1* cDNA rev, 5′-ATTCCATGCCTCG TCCAGTC-3′.

#### Gene expression analysis

Organoids from one to two wells (48-well plate) were collected. RNA was isolated using an RNeasy Micro kit (Qiagen), following the manufacturer's instructions. cDNA was synthesised from 500-1000 ng RNA and diluted 1:40. Per qPCR reaction 1 µl SYBRgreen, 100 nl primer mix (5 µM fwd primer and 5 µM rev primer) and 900 nl cDNA were mixed. For qPCR, the QuantStudio 6 Flex Real-Time PCR System was used. The protocol was composed of a hold stage (2 min at 50°C, 10 min at 95°C) and a PCR stage of 40 cycles (15 s at 95°C, 1 min at 60°C). Relative gene expression was calculated using the ΔΔCt method (Livak and Schmittgen, 2001). RT-qPCR primer sequences were designed using Primer-BLAST. Primers were purchased from TAG Copenhagen A/S. Primer sequences are listed in Table S1.

#### Flow cytometry

Organoids were collected in PBS+0.1% bovine serum albumin (BSA), mechanically dissociated into clumps by pipetting, followed by incubation in TrypLE Express (Gibco, 12605036) for 10-15 min, with pipetting every 5 min. Single cells were washed in PBS+0.1% BSA, filtered through a 35 µm filter and resuspended in PBS with DAPI (0.2 μg/ml). Cells were analysed and/or sorted on a BD FACSAria III sorter.

#### Generation of *Etv1*OE cultures

The *Etv1* gene insert in pLX_TRC311_ETV1 (Addgene plasmid #74981) was cloned into SP170 (PB-TRE-DEST-IRES-BSD) (a kind gift from Steve Pollard's laboratory, Centre for Regenerative Medicine, University of Edinburgh, Edinburgh, UK) using gateway cloning. For the BP reaction pLX_TRC311_ETV1 was linearised with Cla1, and purified DNA (∼250 ng) was mixed with donor vector (pDONR 221 Vector, 150 ng) and 2 µl BP Clonase II mix to a total volume of 10 µl and incubated for 1 h at 25°C. To terminate the reaction, 1 µl Proteinase K (Thermo Fisher Scientific) was added, and samples were incubated for 10 min at 37°C. 1 µl of the BP reaction was transformed into 20 µl Top10 and plated on agar plates with kanamycin. Following overnight incubation, DNA was purified from one colony and used as entry clone for the LR reaction. Entry clone (∼250 ng) was mixed with SP170 destination vector (150 ng) and 2 µl of LR Clonase II enzyme mix to a final volume of 10 µl and incubated for 1 h at 25°C. The reaction was terminated similarly to the BP reaction, and 1 µl was transformed into 20 µl Top10 and plated on agar plates with ampicillin. Following overnight incubation, DNA was purified from one colony, and the construct was sequenced to confirm correct insertion of the *Etv1* gene in the destination vector (pTet-ETV1-blast). Organoids were passaged 5 days prior to electroporation. 2 days before electroporation, R-spondin conditioned medium was replaced with CHIR (ENC medium). 1 day before electroporation, 1.25% DMSO was added to the medium. On the day of electroporation, organoids from 18 wells were collected in PBS+0.1% BSA and dissociated into fragments (three to six cells) by a combination of mechanical dissociation by pipetting and incubation in TrypLE. Fragments were washed twice in optiMEM (Gibco) and resuspended in 200 µl optiMEM. For generation of *Etv1* OE organoids, 100 µl cell suspension was mixed with 4 µg pPB-CAG-rtTA-IRES-Hygro (Addgene plasmid #102423), 8 µg pBase plasmid (a kind gift from Steve Pollard) and 4 µg pTet-ETV1-blast. For generation of control organoids, 100 µl cell suspension was mixed with 4 µg pPB-CAG-rtTA-IRES-Hygro and 8 µg pBase plasmid. Cells were electroporated using a Super Electroporator NEPA21 Type II (Nepagene) with previously published parameters (Fujii et al., 2015). Immediately after electroporation, 400 µl OptiMEM supplemented with ROCKi was added. After 20 min incubation at room temperature, cells were washed once in advanced DMEM/F12 and seeded in 2/3 MG and 1/3 ENC medium. ENC medium supplemented with ROCKi was added after 20 min. 2 days post electroporation, medium was changed to ENR medium with hygromycin (100 µg/ml). Where indicated, organoids were treated with doxycycline (1 µg/ml).

#### Fluorescent *in situ* hybridisation (FISH)

Swiss rolls were made from the small intestine and colon of a C57BL/6 mouse. Tissue was fixed in 4% paraformaldehyde and embedded in OCT (Sakura). FISH was performed using an RNAscope Multiplex Fluorescent Reagent Kit v2 (Advanced Cell Diagnostics) following the manufacturer's instructions. In short, fixed OCT-embedded tissue sections (7 µm) were dehydrated, treated with hydrogen peroxide for 10 min and submerged in boiling target retrieval buffer for 5 min, followed by a 30-min protease treatment at 40°C using HybEZ Oven. Probes directed against *Etv1* and *Pyy* were multiplexed, applied to slides and incubated for 2 h at 40°C in a HybEZ Oven, followed by signal amplification and detection using TSA vivid dyes. Slides were counterstained with DAPI for 30 s and mounted using Prolong Gold Antifade Mountant (Thermo Fisher Scientific). Images were acquired using a Stellaris Confocal Microscope (Leica).

#### Luciferase assay

A G-block spanning a region of 517 bp upstream of *Pyy* covering two putative ETV1 binding sites was designed (PyyProm_WT). Additionally, a G-block in which the two putative ETV1 binding sites were mutated was designed (PyyProm_MUT). For both G-blocks, sequences containing *Hind*III and *Nhe*I restriction cleavage sites were inserted at the beginning and end of the genomic sequence, respectively. G-blocks were purchased from IDT, and sequences are listed in Table S2. G-blocks were cloned into the pGL4.23 (*luc2*/minP) vector (Promega) using restriction cloning. pGL4.23 (1 µg) and G-blocks (100 ng) were cut with *Hind*III and *Nhe*I by incubation at 37°C for 1 h, and DNA was purified using the MinElute PCR Purification Kit (Qiagen). Cut pGL4.23 (50 ng) and G-blocks were mixed and ligated by incubation with T4 DNA ligase for 10 min at room temperature followed by 10 min heat inactivation at 65°C. Ligated plasmids were transformed into One Shot^®^ TOP10 Chemically Competent E. coli (Invitrogen), and DNA was isolated from overnight cultures using a Plasmid Mini Kit (Qiagen). Correct insertion of G-blocks was confirmed by Sanger sequencing. HEK293 cells were seeded in a 96-well plate, and, upon reaching 80% confluency, cells were transfected with 10 ng pGL4.23 vector with no DNA inserted, PyyProm_WT inserted and PyyProm_MUT inserted together with 10 ng pPB-CAG-rtTA-IRES-Hygro, 10 ng pBase plasmid, 10 ng pTet-ETV1-blast and 1 ng pRL Renilla Luciferase Control Reporter Vector (Promega) using Lipofectamine 2000. 1 day post transfection, cells were treated with doxycycline (1 µg/ml). On day 2, Luciferase activity was measured using the Dual-Luciferase^®^ Reporter Assay System (Promega) following the manufacturer's instructions. Luminescence was measured using a SpectraMax^®^ iD3 Multi-Mode Microplate Reader (Molecular Devices).

#### scRNA-seq

Control and *Etv1* mutant organoids were isolated on day 5 after passaging and dissociated into single cells similarly to as described for flow cytometry. After incubation in TrypLE and washing in PBS+0.1% BSA, cells were resuspended in 100 µl PBS+0.1% BSA, and 1 µl hashtaq oligo (HTO) antibody was added (0.5 µg) pr sample for multiplexing (A0308-A0312, BioLegend). Cells were incubated for 20 min on ice, washed twice in PBS+0.1% BSA, and incubated in 200 µl PBS+0.1% BSA with DAPI (0.2 μg/ml) for 5-10 min on ice. Finally, cells were filtered through a 35 µm filter and resuspended in 200 µl ultra-clean 1% BSA, and 6000 events per sample were sorted into a low-binding 1.5 ml Eppendorf tube with 3 µl ultra-clean 1% BSA on a BD FACSAria III sorter. scRNA-seq libraries were prepared using the Chromium Next GEM Single Cell 3′ GEM, Library & Gel Bead Kit v3.1 according to the manufacturer's instructions. Some additional steps required to generate HTO libraries were included, as we have previously described (Hansen et al., 2023). cDNA was amplified with ten PCR cycles, and sample-indexing PCR was performed with 13 cycles. The HTO cDNA and endogenous cDNA libraries were diluted to 2 nM in EB buffer (Qiagen) and pooled to a final library (15% HTO and 85% endogenous cDNA). For sequencing, the NovaSeq 6000 S1 Reagent Kit v1.5 (100 cycles) was used. The pooled library was further diluted to a concentration of 400 pM and sequenced with a Novaseq 6000 sequencer.

#### scRNA-seq analysis of *Etv1* mutant organoids

FASTQ files were generated from raw base call files using Cell Ranger's mkfastq function [10X Genomics Cell Ranger v 6.1.0 (Zheng et al., 2017)]. For quantification of reads, the salmon alevin v/feature barcodes pipeline (Salmon, v 1.4.0; Patro et al., 2017) was used. First, an index of reference sequences and HTO sequences was built. For the reference sequence index mm10 index was used. Then salmon alevin (Salmon, v 1.4.0) was used to pseudo-quantify RNA and HTO reads. The remaining analysis was performed in R using the Seurat package (v 4.3.0; Hao et al., 2021). The HTO and RNA count matrices were loaded into R, and a Seurat object containing cells found in both matrices was generated. Data were normalised using the NormalizeData() function with method set to CLR and margin set to 1. HTOs were demultiplexed with the HTODemux() function from the Seurat package using default settings. Following demultiplexing of HTOs, cells with no HTO and cells with more than one HTO were excluded. Because removal of cells with more than one HTO will eliminate the vast majority of duplets, no additional doublet filtering was performed. The remaining cells were filtered to exclude cells with more than 7.5% of counts originating from mitochondrial features and/or less than 1000 total features. Data were log normalised, and the CellCycleScoring() function was used to assign S and G2/M scores using the S and G2/M genes provided by Seurat. Data were scaled using Scaledata(), and differences caused by cell cycle states were removed. The 2000 most variable features were identified and used to perform PCA and UMAP. For clustering, FindNeighbors() using the first eight principal components (PCs) and FindClusters() using a resolution of 0.3 were used. Cell clusters were annotated based on expression of canonical marker gene expression.

#### scRNA-seq analysis of published datasets

The mouse small intestinal scRNA-seq dataset was downloaded from https://singlecell.broadinstitute.org/single_cell/study/SCP44/small-intestinal-epithelium. We performed batch correction using the Seurat functions FindIntegrationAnchors() and IntegrateData(), as, upon unsupervised clustering, we observed a batch effect caused by the individual mice from which cells originated (Fig. S1A,B) (Stuart et al., 2019). To visualise the data before and after batch correction, data were first scaled, after which the 2000 most variable features were identified and used to perform PCA and UMAP. To identify neighbours, the first 15 PCs were used. For analysis of EECs, the dataset was subset to only include this cluster (original annotation). Again, data were scaled, and the 2000 most variable features were identified and used to perform PCA and UMAP, with the first 15 PCs being used to identify neighbours. Analysis of the mouse organoid dataset (Hansen et al., 2023) was performed in a similar manner, with the exception that batch correction was not necessary.

## Supplementary Material

10.1242/dmm.052610_sup1 Supplementary information
