# Supplementary material for: ETV1 is a key regulator of enteroendocrine PYY production
Source: Dis Model Mech. 2025 Dec 12;18(12):dmm052610. doi: 10.1242/dmm.052610 (PMC12746708; doi:10.1242/dmm.052610)
Supplement: Supplementary information [file dmm-18-052610-s1.pdf]

Supplementary figure 1

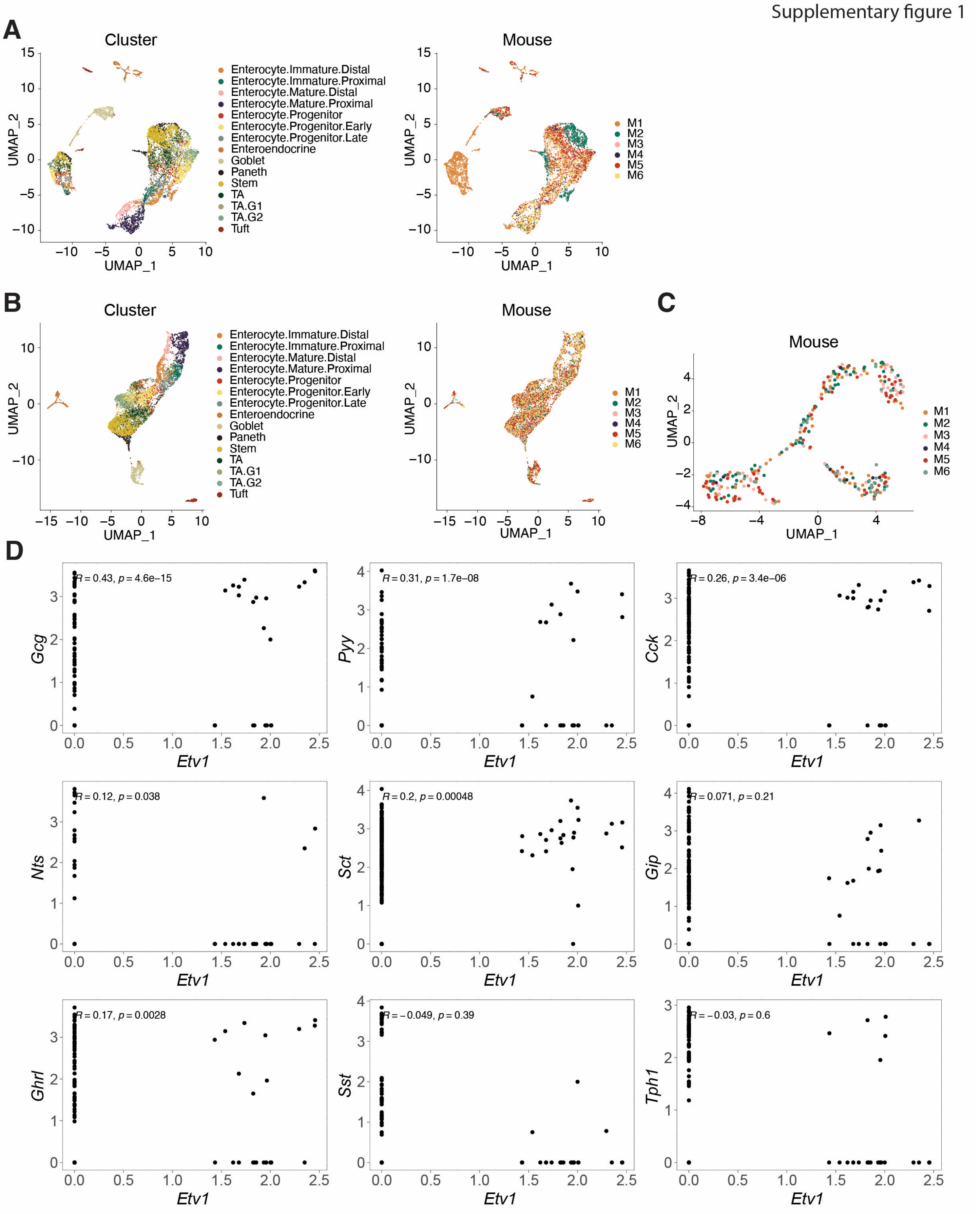

Fig. S1. Analysis of scRNA-seq data from mouse small intestine. A+B) UMAP plot showing cells annotated by cluster (Annotation from Haber et al. [43]) and mouse (A) before and (B) after batch correction. **C)** UMAP plot of subclustered EECs annotated by mouse. **D)** Correlation plots showing the correlation between *Etv1* expression and EE hormones.

Disease Models & Mechanisms • Supplementary information

Supplementary figure 2

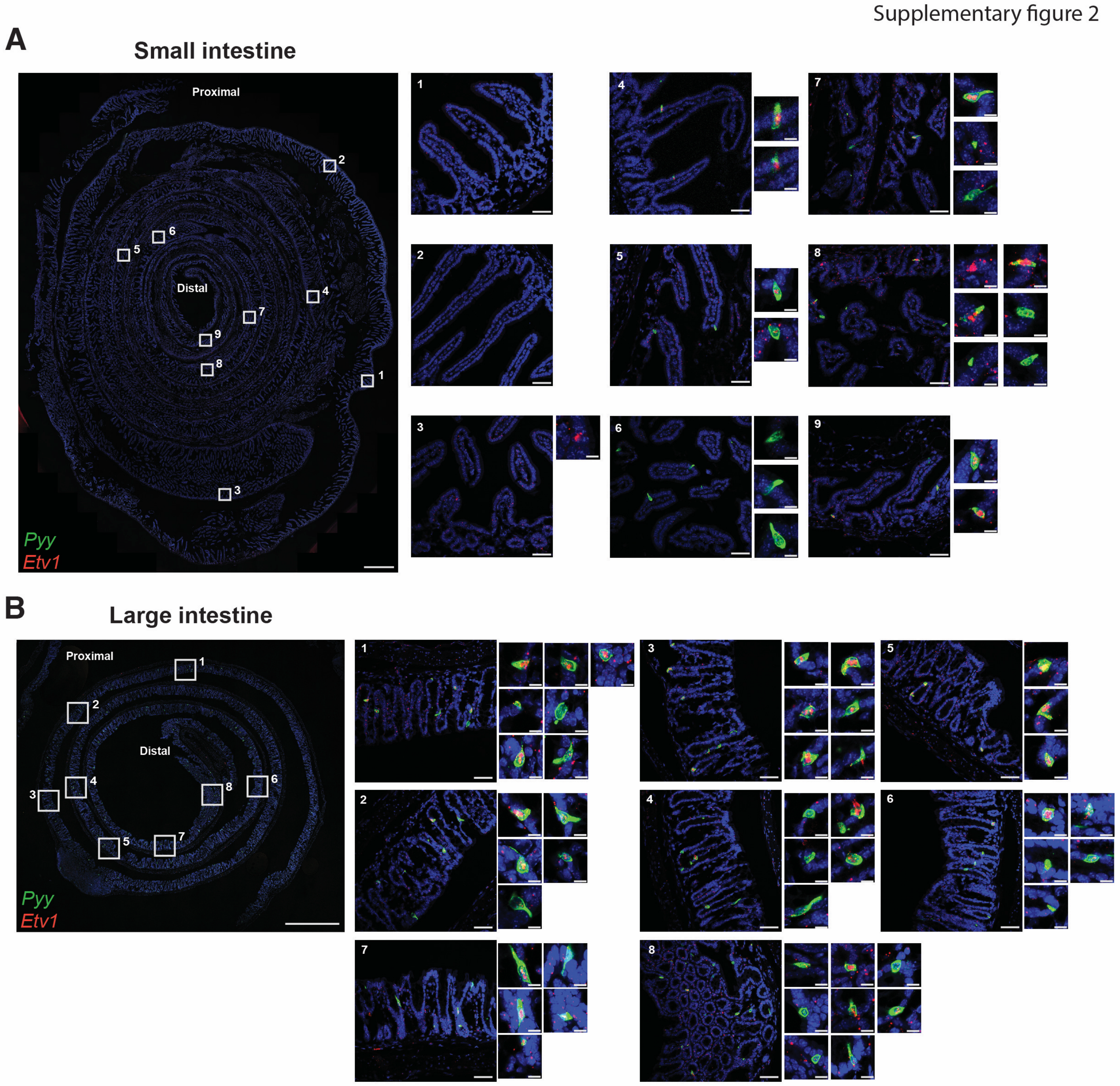

Fig. S2. ***Etv1*** **and** ***Pyy*** **co-expression in the small and large intestine.**

A) Flourescent in situ hybridization showing presence of Etv1 (red) and Pyy (green) transcripts in mouse small intestine. Scalebar = 1mm. Scalebar (inserts) = 50 µm. Scalebar (small inserts) = 10 µm. **B)** Flourescent in situ hybridization showing presence of Etv1 (red) and Pyy (green) transcripts in mouse large intestine. Scalebar = 1mm. Scalebar (inserts) = 50 µm. Scalebar (small inserts) = 10 µm.

Disease Models & Mechanisms • Supplementary information

Supplementary figure 3

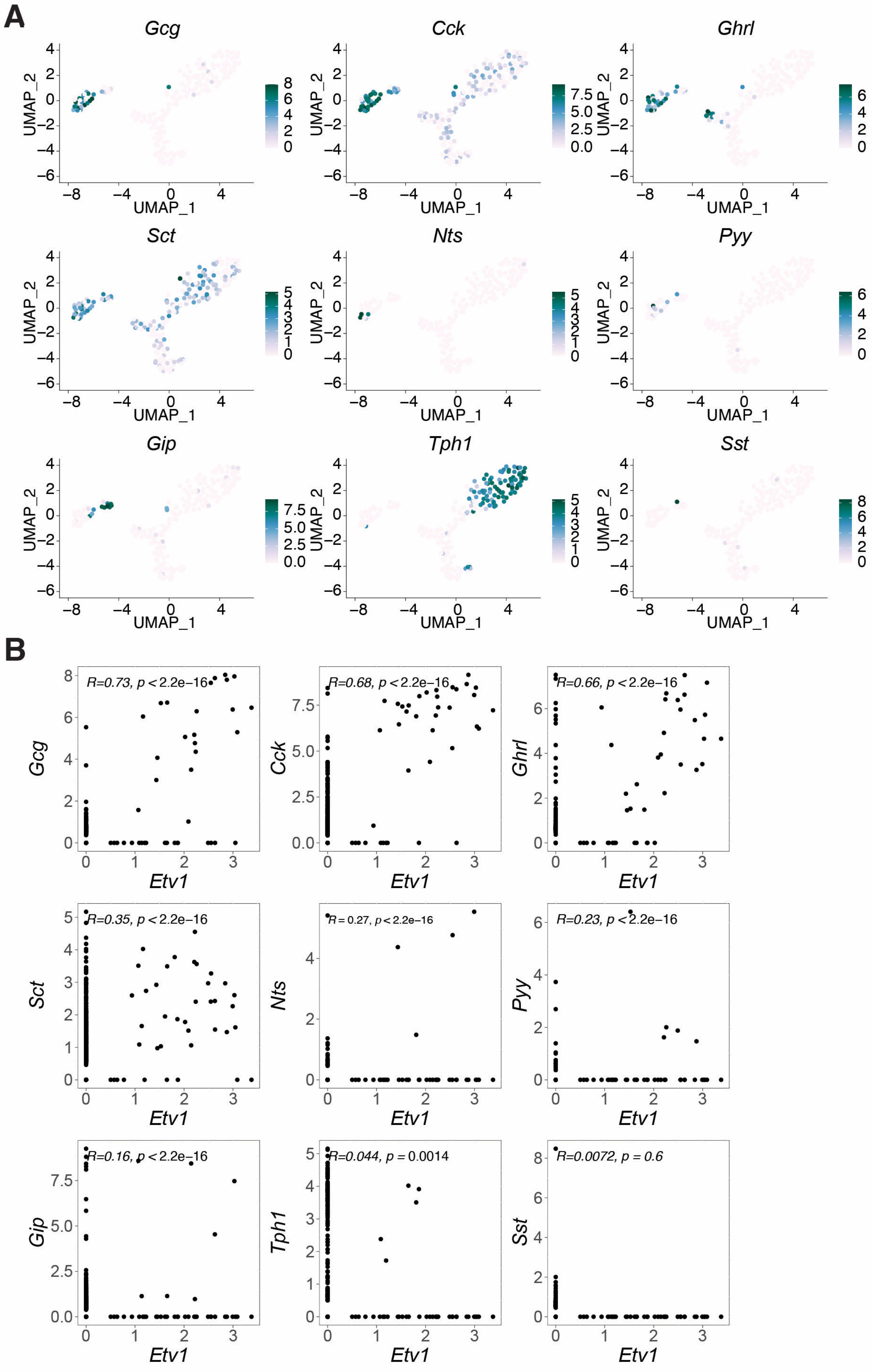

Fig. S3. Analysis of scRNA-seq data from mouse small intestinal organoids.

A) UMAP plots showing expression levels of EE hormones in EECs from organoids. **B)** Correlation plots showing the correlation between *Etv1* expression and EE hormones. **C)** Correlation plots showing the correlation between *Etv1* and *Ghrl* expression, combined with expression of *Gcg* (top) and *Cck* (bottom).

Supplementary figure 4

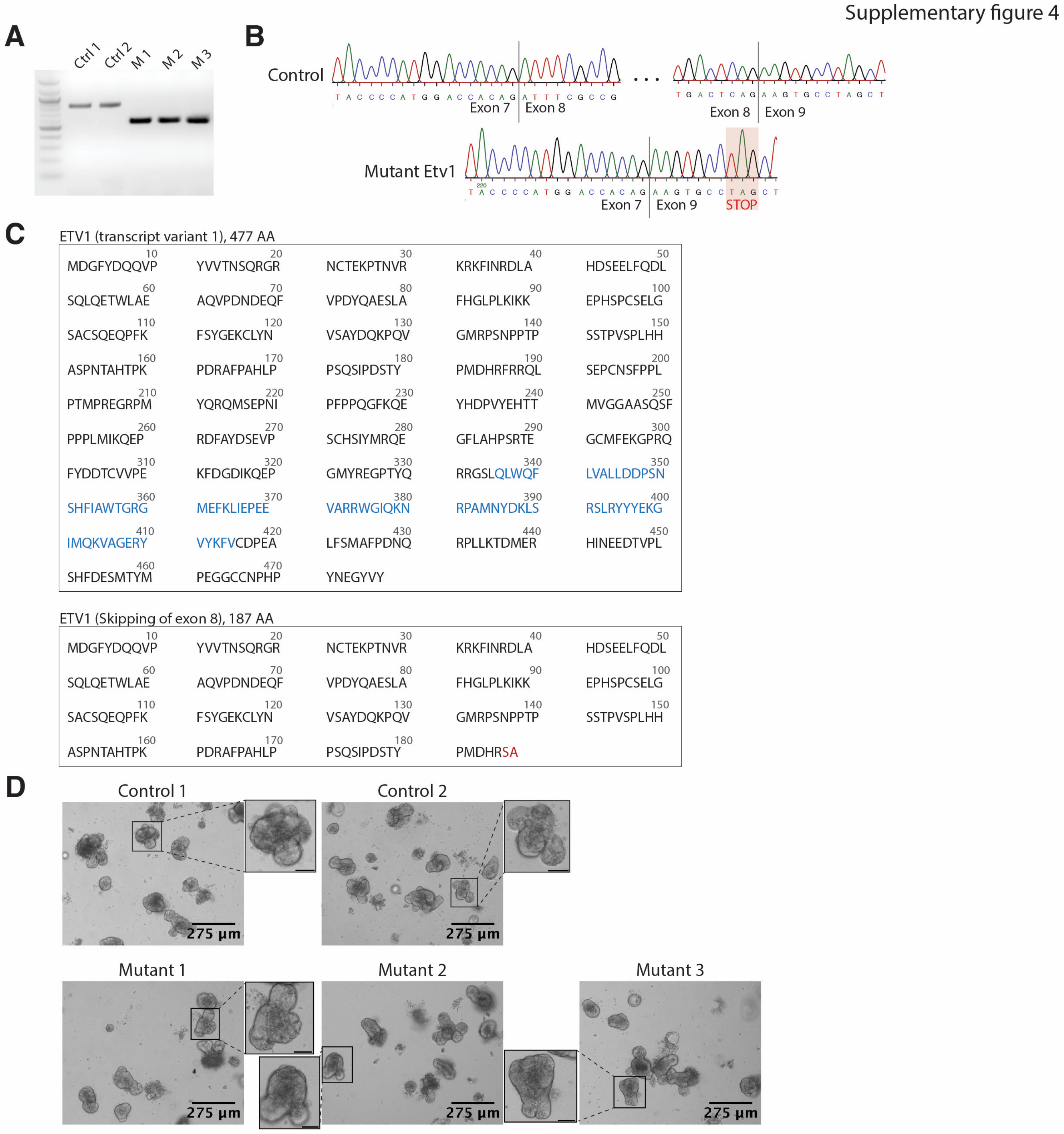

Fig. S4. Characterization of *Etv1* mutant cultures.

A) Agarose gel electrophoresis of PCR products following amplification of *Etv1* cDNA in control and *Etv1* mutant organoid lines. **B)** Results from sanger-sequencing of purified PCR products show exon skipping of exon 8 in *Etv1* mutant organoid lines. Exon skipping introduces a premature stop codon (marked in red) **C)** Amino acid sequence of Etv1 transcript variant 1 (top) and truncated ETV1 protein following exon skipping of exon 8 (bottom) in *Etv1* mutant organoids. Blue indicates the ETS DNA binding domain. Red indicates mutated amino acids prior to the premature stop codon. **D)** Bright-field images showing control and *Etv1* mutant clonal organoid cultures. scalebars = 275 µm. Scalebars in inserts = 125 µm.

Disease Models & Mechanisms • Supplementary information

Supplementary figure 5

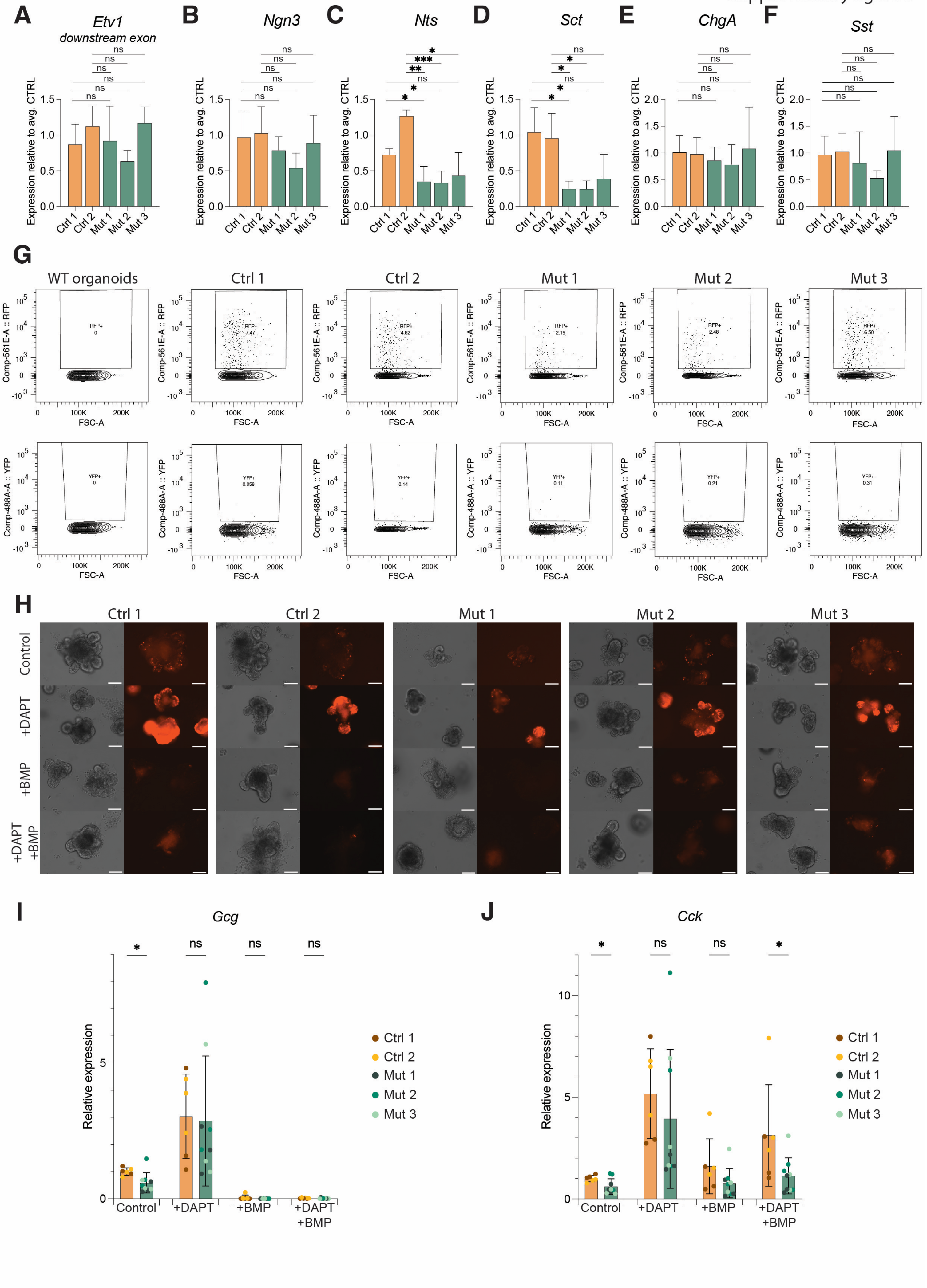

Fig. S5. Expression of *Neurog3* and EE hormones in *Etv1* mutant organoid cultures.

A) Expression of *Etv1* in control and *Etv1* mutant organoid cultures using a primer-pair located downstream of exon 8. Expression is normalized to expression of *Gapdh*. Error bars indicate SD (n=3). Significance was evaluated with a student's t-test, ns = not significant. **B-F)** Expression of **B)** *Ngn3*, **C)** *Nts*, **D)** *Sct*, **E)** *ChgA,* and **F)** *Sst* in control and *Etv1* mutant organoid cultures. Expression is normalized to expression of *Gapdh*. Error bars indicate SD (n=3). Significance was evaluated with a student's t-test, ns = not significant, *P<0.05, **P<0.01, ***P<0.001. **G)** Flow cytometry–based contour plots of Neurog3-RFP and Gcg-Venus expression in control and *Etv1* mutant organoid cultures. H) Bright-field and fluorescent images showing Neurog3-RFP in control and Etv1 mutant organoid cultures treated for 3 days with or without 10 µg DAPT and/or 20 ng/ml BMP-4. Scale bar = 100 µm. **I-J)** Expression of **I)** *Gcg* and **J)** *Cck* in control and *Etv1* mutant organoid cultures treated for 3 days with or without 10 µg DAPT and/or 20 ng/ml BMP-4. Significance was evaluated with a student's t-test, *P<0.05, **P<0.01, ***P<0.001, ****P<0.0001.

Disease Models & Mechanisms • Supplementary information

Supplementary figure 6

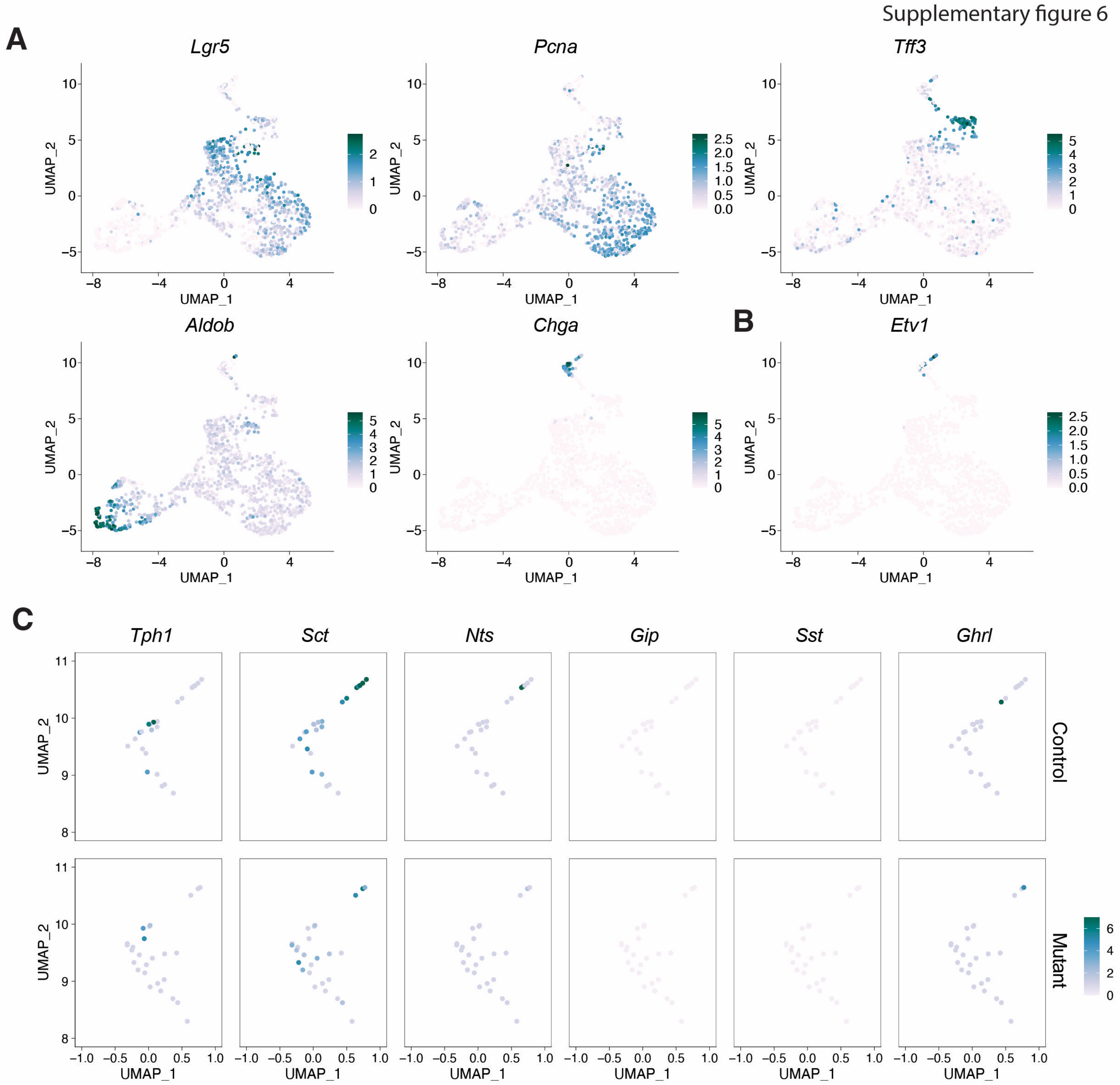

Fig. S6. Analysis of scRNAseq-data from *Etv1* mutant organoid cultures.

A) UMAP plots showing expression levels of canonical cell type and proliferation marker genes in cells from control and *Etv1* mutant organoids. *Lgr5* marks ISCs, *Pcna* marks proliferating cells and is enriched in TA-cells, *Tff3* marks goblet cells, *Aldob* marks enterocytes and *Chga* marks EECs **B)** UMAP plot showing expression levels of *Etv1* in cells from control and Etv1 mutant organoids. **C)** UMAP plots showing expression levels of *Tph1*, *Sct*, *Nts*, *Gip*, *Sst,* and *Ghrl* in EECs from control (top) and *Etv1* mutant (bottom) organoids.

Disease Models & Mechanisms • Supplementary information

Supplementary figure 7

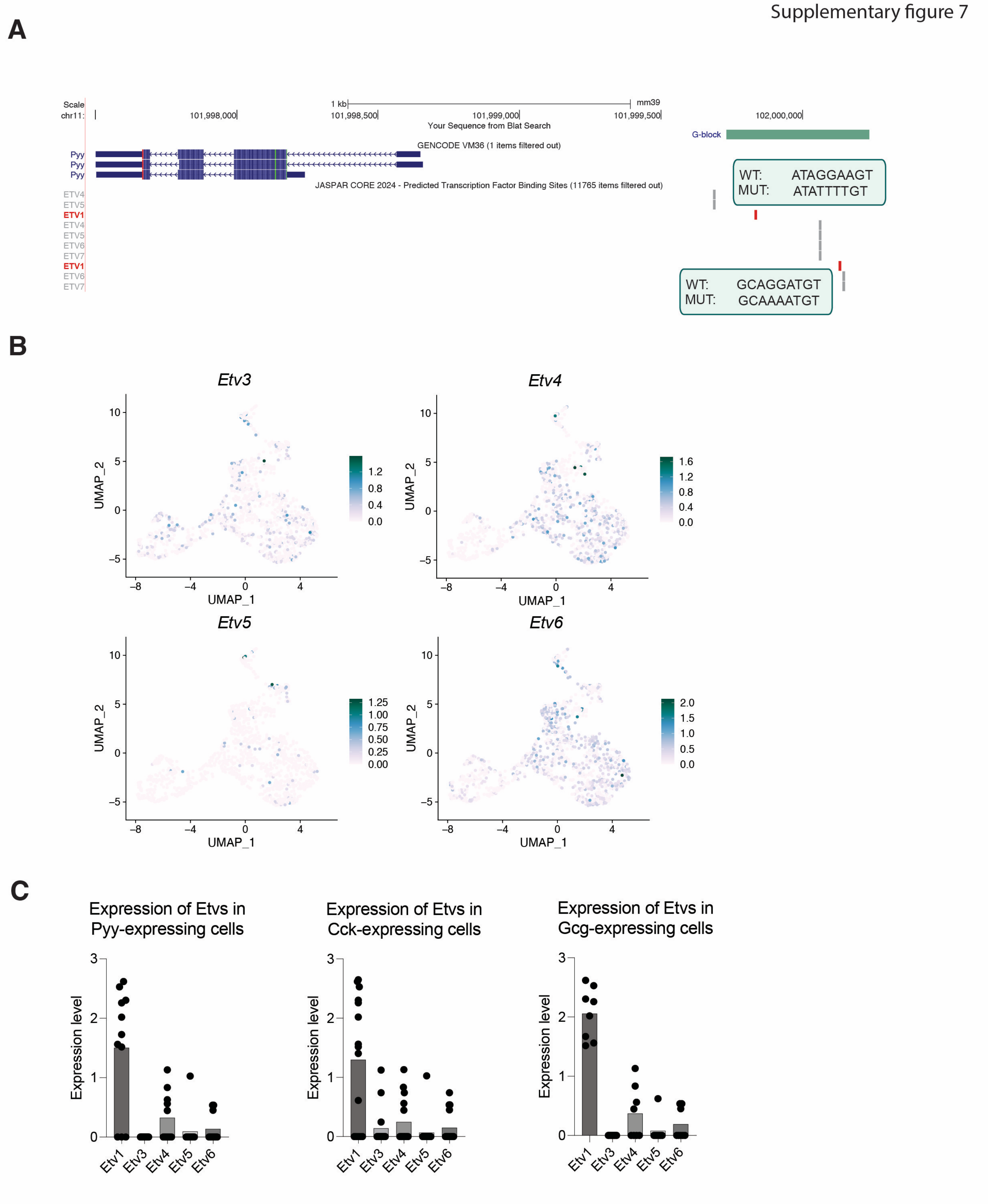

Fig. S7. ETV binding sites upstream of *Pyy* and expression of the *Etv* family of transcription factors in mouse small intestinal organoids.

A) Screenshot downloaded from http://genome.ucsc.edu showing putative Etv binding sites upstream of *Pyy.* A G-block spanning a region of 517 bp covering two putative Etv1 binding sites was designed (PyyProm_WT). Additionally, a G-block in which the two putative Etv1 binding sites were mutated was designed (PyyProm_MUT). **B)** UMAP plots showing expression levels of Etv3/4/5/6 in cells from control and *Etv1* mutant organoids. Etv2/7 are not expressed. **C)** Expression of Etv1/3/4/5/6 in cells expressing Pyy, Cck and Gcg, respectively.

Disease Models & Mechanisms • Supplementary information

Table S1. qPCR primer sequences

Target	Forward primer	Reverse primer
Cck	CCCCAATGTGAAATCTGTCC	GGTCTGGGAGTCACTGAAGG
Etv1 primer1	CCCAGAGATTTTGCATATGACTC	GCCTTCTGTTCTGCTTGGA
Etv1 primer2	AAGGGTCCCAGGCAGTTCTA	GGGTCCTTCCCGGTACATTC
Gapdh	TGTTCCTACCCCCAATGTGT	TGTGAGGGAGATGCTCAGTG
Gcg	TGTCTACACCTGTTCGCAGC	TCTGTGTCTTGAAGGGCGTG
Ngn3	AGTTCCAATTCCACCCCACC	AGTCACCCACTTCTGCTTCG
Nts	CTGGTGTGCCTGACTCTCCT	TCACATCTTCTTCTGAATCTGAGC
Pyy	GCAGCGGTATGGAAAAAGAG	GTCGCTGTCGTCTGTGAAGA
Sct	GATGGGTCCCTGTCTCTCTG	GCCTGGTTGTTTCAGTCCAC
Sst	GGCTGCCACCGGGAAACAGG	AGCTTTGCGTTCCCGGGGTG
Chga	GAACAGCCCCATGACAAAAG	TCGGAGATGACTTCCAGGAC

Table S2. G-block sequences

Name	Sequence
**PyyProm_WT**	**cccagtgcaagtgcaggtgccagaacatttctctggcctaactggccggtacctgagc**<br>**tcGCTAGC**GACCTCCAGATACAGGCTCCCTAAGGAGAACTGTGTCT<br>CTCTTTTCTCCAAGGCTGGGCTACATGTCCTCTCATCCTGATCTTTT<br>CCACTTCCGTGCTCAGCAGGATGTCCCAGTTCTTCTTCTGTTACAG<br>GGGGGCCCCTGTGGGACCTGGCTGGCTGATAAGGATCCATTTCCG<br>GAGAGTGCAGGTGACTGAACCCATAAAAAAGGTGATTGAAGAAGCC<br>CTGGATGCTCCATCTCAGGTGAGTTCTAAAAATAGCATCCACAGCC<br>ATCTGTCCTGGGAATTGGAAGGGCTGGCCCAGAACTAGGGGCCTG<br>GGCAAGATGGTTCTCCCTGTTTCCATGGGCTAGAAGTGGTGGAGA<br>CTCTGGCAGGGCTGGGAGAAGGACCCAGAAAGGGTTGGACTTCCT<br>ATGGTCCTCTCTTCTTGGTTAAGCAGGTGGGTCTGATAGGGCTCTT<br>CCCCATGGGGCTGGTGGTCTGGGGTCCTTCCCTCTCTCAGCCAATC<br>AGAGTAGGCT**AAGCTTagacactagagggtatataatggaagctcgacttcca**<br>**gcttggcaatccggtactgttggtaaagccacc**
**PyyProm_MUT**	**cccagtgcaagtgcaggtgccagaacatttctctggcctaactggccggtacctgagc**<br>**tcGCTAGC**GACCTCCAGATACAGGCTCCCTAAGGAGAACTGTGTCT<br>CTCTTTTCTCCAAGGCTGGGCTACATGTCCTCTCATCCTGATCTTTT<br>CCACTTCCGTGCTCAGCAAAATGTCCCAGTTCTTCTTCTGTTACAGG<br>GGGGCCCCTGTGGGACCTGGCTGGCTGATAAGGATCCATTTCCGG<br>AGAGTGCAGGTGACTGAACCCATAAAAAAGGTGATTGAAGAAGCCC<br>TGGATGCTCCATCTCAGGTGAGTTCTAAAAATAGCATCCACAGCCA<br>TCTGTCCTGGGAATTGGAAGGGCTGGCCCAGAACTAGGGGCCTGG<br>GCAAGATGGTTCTCCCTGTTTCCATGGGCTAGAAGTGGTGGAGACT<br>CTGGCAGGGCTGGGAGAAGGACCCAGAAAGGGTTGGACAAAATAT<br>GGTCCTCTCTTCTTGGTTAAGCAGGTGGGTCTGATAGGGCTCTTCC<br>CCATGGGGCTGGTGGTCTGGGGTCCTTCCCTCTCTCAGCCAATCAG<br>AGTAGGCT**AAGCTTagacactagagggtatataatggaagctcgacttccagct**<br>**tggcaatccggtactgttggtaaagccacc**

Disease Models & Mechanisms • Supplementary information
